# The Effect of Solar Light on Vegetable and Animal Fæcula, &c.

**Published:** 1860-03

**Authors:** Niepce De Saint Victor, Lucien Corvisart


*The Effect of Solar Light on Vegetable and Animal Fæcula, &c.* By NIEPEC DE SAINT VICTOR and LUCIEN CORVISART.

1. Solar light, by an action peculiar to itself, modifies and transforms certain amylaceous substances and some of their derivatives.

2. This action alone, when prolonged, is capable of transforming a solution of pure starch into dextrine and sugar. But at first light modifies entirely the nature of starch, converting it into a substance resembling inuline, as obtained from the alder, colchicum, &c., in which condition it is, while cold, entirely insensible to the action of iodine, but which differs from inuline, since it will not reduce the salts of copper and silver in the presence of ammonia. It does not alter the plane of polarization. This change may be effected in six hours of good exposure, during the months of July and August; but oftener twelve or eighteen hours of exposure are required to produce the complete change. A solution of starch, containing half a thousandth, although exposed in the same place at the same time, and with the same temperature, if protected by a dark covering, will undergo no change, so that a few drops of the latter solution would produce a deep blue in a mixture of the former and iodine.

3. This transforming action is impeded by one per cent. of the lactate or citrate of iron in the solution, and by one-half per cent. of nitric and tartaric acid; and it is completely prevented by corrosive sublimate.

4. It is facilitated by the potassa-tartrate of iron, nitrate of uranium, and oxalic acid.

5. Whatever they may be, simple or only primordial, primitive or secondary, the cause of these changes is the *luminous principle.*

6. Dextrine is, however, more an artificial than a natural product. That obtained by diastase, not reducing the reagents of Barreswil and Fehling, undergoes no change from the action of light.

7. Cane sugar undergoes no change. Oxalic acid, one of the derivatives of starch, in solution along with nitrate of uranium, may be boiled, and then kept for forty hours in a stove at 40° C., without the disengagement of a bubble of gas, provided the experiment is performed in the dark. As soon as light, even diffused and clouded, is admitted by raising the cover of the stove, the mixture will begin to exhibit changes. A good exposure of an hour will produce abundance of carbonic oxide, so rapid, indeed, as almost to produce effervescence.

8. In accordance with the direct experiments we have made, animal fæcula (glucogenic material) is more rapidly and abundantly converted into sugar in the light than in darkness, although nitrate of uranium will even hinder this action.

9. Animal fæcula remains in the liver of frogs, without becoming sugar, during the whole of winter. The greatest quantity of sugar in their livers coincides with the period of the ripening of fruits, the end of June, and the months of July and August. The glucogenic matter may be fixed in the liver, just as starch is in tubers or grains. If frogs are kept entirely from the light, no sugar will be formed. We might explain, in this way, how the large amount of glucogenic matter in the cutaneous tissue of the fœtus disappears from this tissue soon after birth, by its sudden passage from obscurity into light.

10. It must also be recollected that although a small quantity of light is required, yet its action must be aided by the presence of certain salts or ferments, and that, in most animals and man, the *amylogenic* as well as the *glucogenic* functions experience no hibernal cessation from work.

11. The actions of light, already described, are generally slow.

12. Hence, if, without augmentation of light, certain substances, on the one hand, double, treble, or sextuple the effects of solar action in the formation of animal or vegetable sugar; if, on the other hand, without diminution of solar intensity, certain others destroy or hinder the change, as in starch, which arises from solar action, it cannot be denied but that exact studies directed to this end would be very useful, as well for vegetable physiology as for agriculture, and possibly also for medicine. It is only necessary to cite diabetes and the influence of exposure to sunlight on scrofula.—*Gazette Hebdomadaire*, September, 1859. L. H. S.

